# Resumption of sport after spinal fusion for adolescent idiopathic scoliosis: a review of the current literature

**DOI:** 10.1007/s43390-021-00330-6

**Published:** 2021-03-23

**Authors:** Francesca Barile, Alberto Ruffilli, Marco Manzetti, Michele Fiore, Alessandro Panciera, Giovanni Viroli, Cesare Faldini

**Affiliations:** Department of Biomedical and Neuromotor Science - DIBINEM, 1st Orthopaedic and Traumatologic Clinic, IRCCS Istituto Ortopedico Rizzoli, University of Bologna, Via Giulio Cesare Pupilli 1, 40136 Bologna, Italy

**Keywords:** Scoliosis and sport, Adolescent idiopathic scoliosis, Return to sport

## Abstract

**Background:**

Adolescent idiopathic scoliosis (AIS) is a frequent disorder. Since patients with AIS are typically as active as age-matched controls and post-operative reduction in physical activity has detrimental effects on their well-being, return to sport (RTS) is an important perioperative concern. Aim of the present study is to review the literature concerning return to sport after spinal fusion for AIS.

**Methods:**

This work was carried out in accordance with Preferential Reporting Items for Systematic Reviews and Meta-analyses (PRISMA) guidelines. The search was carried out in December 2020. Only peer-reviewed randomized controlled trials (RCTs), retrospective studies (RS), retrospective case series (RCS) and perspective cohort studies (PCS) were considered for inclusion.

**Results:**

Six studies were included; only one of them was prospective. All the authors reported a time to RTS ranging between 6 and 18 months. Between 28 and 36.6% of all patients changed sport, choosing lower impact activities, mostly due to loss of flexibility of the spine. No complications due to return to play were noted.

**Conclusion:**

According to current evidence, patients who received spinal arthrodesis for AIS can safely return to any sport, even those that require extreme levels of spinal and pelvic movements such as gymnastics and golf. As there is little evidence, however, of the spinal loading that occurs during such movements, there is a lack of scientific evidence-based recommendations or guidelines surgeons and other health care providers can follow. Prospective comparative studies are needed to investigate these biomechanical and clinical issues.

**Level of evidence:**

Level III.

## Introduction

Adolescent Idiopathic Scoliosis (AIS) is a frequent musculoskeletal disorder [1]; although 2–3% of the adolescent population is affected, less than 10% of these patients require a surgical intervention of spinal fusion [1]. Since patients with a diagnosis of AIS are typically active as age-matched controls [2] and post-operative reduction in physical activity can have detrimental effects on their health and well-being [3], return to sport (RTS) is often an important perioperative concern for the patients and families [2]. Nevertheless, current guidelines for postoperative athletic participation are derived from expert opinion and no evidence-based recommendations exist regarding tailored rehabilitation [4] nor the timing of return to sport (RTS) after spine surgery for AIS. Aim of this study is to review the available literature concerning return to sport after spinal arthrodesis for Adolescent Idiopathic Scoliosis.

## Materials and methods

The present work was carried out in accordance with Preferential Reporting Items for Systematic Reviews and Meta-analyses (PRISMA) guidelines.

### Eligibility criteria

Only peer-reviewed publications were considered for inclusion. Studies were included if they involved patients who underwent spinal fusion to correct AIS and if they described their RTS after surgery. Only articles who meet the PICO criteria on systematic reviews (Population, Intervention, Comparison and Outcomes) were included.

The type of study considered for inclusion were randomized controlled trials (RCTs), retrospective studies (RS), retrospective case series (RCS) and perspective cohort studies (PCS), while case reports, literature reviews and meta-analyses were excluded. In vitro or biomechanical studies and cadaver experiments were also excluded. According to the reviewer’s language capabilities, considered studies were those written in English and Italian.

### Information sources and search strategy

Electronic research to identify eligible studies was performed using online databases including PubMed-MEDLINE, the Cochrane Central Registry of Controlled Trials and Embase Biomedical database by two reviewers (MM and FB). The literature search was carried out in December 2020. Search terms included were “adolescent idiopathic scoliosis”, “return to sport” and “physical activity”.

### Study selection

**Fig. 1:**
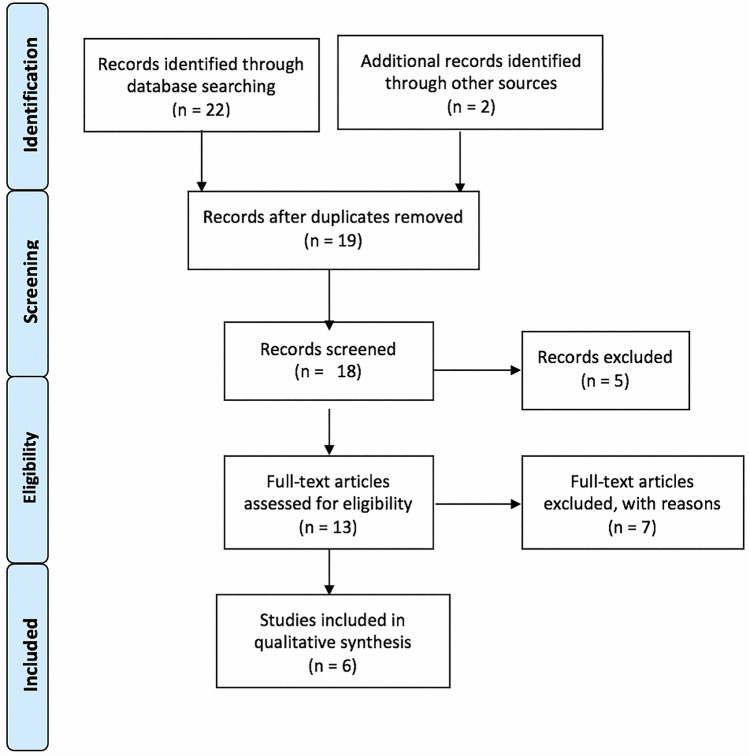
PRISMA flowchart illustrating the search strategy and number of records screened and included

Once the studies eligible for inclusion had been retrieved, the full text of articles was obtained and evaluated. A manual search through the bibliography of each of the relevant articles was also performed to identify potentially missed eligible papers. Duplicates were removed. The study selection process carried out in accordance with the PRISMA flowchart [5, 6], is shown in Fig. 1.

### Data collection process

All the included studies were analysed, and the following data were extracted and are summarized in Table 1: study design, number of patients, mean age, average number of levels fused, mean follow-up, mean time to RTS, correlation between surgery and RTS.

**Table 1:** Analysis of all available included studies concerning the relationship between surgical correction of adolescent idiopathic scoliosis and return to sport

Study	Study design	Patients (*n*.)	Mean age (yrs)	Average *n*. level fused	Mean follow-up (yrs)	Mean return to school (months)	Mean return to sport (months)
Fabricant et al. (2012) [7]	RS	42	15.1	9,9	5.5	–	7.4
Tarrant et al. (2014) [8]	PCS	77	15.04	12	1	2.5	18
Lonner et al. (2014) [9]	RS	38	14.2	9.5	1.5	–	6–12
Perkins et al. (2017) [10]	RS	41	–	–	–	2.5	15
Sarwahi et al. (2015) [11]	RS	82	16	12	–	3	6–12
Sarwahi et al. (2018) [12]	RS	95	15	11	2	1–6	7–12

*N* number, *Yrs* years, *RTS* return to sport

## Results

### Included studies

According to the search performed, a total of six studies [7–12] met the inclusion criteria and were included for review. Of these studies, one was a PCS [8] and five were RS [7, 9–12]. In addition to the analysis of the clinical documents, five [7, 9–12] of the authors of the included studies administered post-operative questionnaires to all their patients: Fabricant et al. [7]used the SRS-22 [13]; Lonner et al. [9] administered the SRS-22, the Activity Questionnaire for Adults and Adolescents (AQuAA)[14] and a personalized questionnaire; Sarwai et al. chose a 24-questions survey in 2015 [11] and the Sport Activity Questionnaire (SAQ) in 2017 [12]; Perkins et al. [10] used a non-validated telephone questionnaire. The studies included in the search reported data on a total of 375 patients. Medium follow-up ranged from 1 to 5.5 years and medium age at surgery ranged from 14.2 to 16 years.

Included studies are not homogeneous (or lacking data) in Lenke distribution of the curves and preoperative Cobb angle.

### Type of surgery

All patients evaluated in the included studies underwent surgical fusion of the thoracic or thoracolumbar spine. Either posterior (Posterior Spinal Fusion, PSF) or combined (anterior plus posterior) approaches were performed; two studies [7, 8] included only patients who received PSF, one study [9] included 36 patients who received PSF and 2 patients who received a combined approach. The others [10–12] did not report any data concerning surgical approach. None of the studies reported data concerning surgical technique (pedicle screws or hooks, high or low-density constructs).

Regarding the number of fused levels, the included studies reported a mean value between 9.5 [7] and 12 [8, 11]. Only two studies reported the mean Lower Instrumented Vertebra (LIV): L2 in Fabricant et al. [7] and L3 in Tarrant et al. [8] cohort.

### Return to school and to physical activity

Four [8, 10–12] of the included studies analysed the mean time until return to school: they all reported it to be between 10 and 12 weeks. Considering return to sport, the authors of all the included studies reported a time ranging between 6 months (50–60% of the patients [8, 11, 12]) and 18 months (> 90% of the patients [8, 10–12]); no statistically significant difference was reported between contact and non-contact activities. The percentage of patients who resumed sport with equal or greater performance (compared to preoperative condition) varied from 31.7% [12] to 80.6% [9]. However, between 28% [7] and 36.6% [10] of all patients changed sport, choosing lower impact activities. Infact, about patient-perceived physical potential in sport, one study [9] reported no significant changes, whilst two studies [7, 10] reported a list of reasons for the choice of changing sport and/or for the decline in the level of athletic participation: the most common was the loss of flexibility, followed by pain, fear of injuries and deconditioning. Moreover, Lonner et al. [9] and Perkins et al. [10] reported a significantly reduced time spent in the sport after surgery: the mean hours per week dropped from a range of 6–10 to a range of 2–4.

No complications due to return to play were noted by any of the three authors who addressed the problem [7, 11, 12]. The only complication that was directly related to RTS was an instrumentation pullout without neurologic deficit in one patient who went snowboarding 2 weeks postoperatively [23].

Four [7, 9, 11, 12] of the six included studies reported some variables to be significantly associated with patients’ later RTS and/or lower performance: a more distal Lower Instrumented Vertebra [7, 9–11], the SRS-22 outcomes [7], the preoperative Lenke value [7], younger age (mean age 14.7 years) [11]. The other two studies [8, 12] found no factors to delay return to any level of sports.

## Discussion

While there is an agreement that individuals who underwent spinal fusion for AIS can perform maximal-effort sport movements without inducing an acute injury [15–21], some controversy still exists surrounding when the patients should return to sports after surgery [21].

Surgeons and clinicians are often cautious, to avoid a patient from undergoing spinal trauma during movements such as rotations and bending [22]. Obviously, their approach towards allowing a return to sports activity is strongly influenced by the surgical strategy that has been chosen: above all, length of the fusion and type of implants. In 2002, 271 Scoliosis Research Society members participated in a survey on sports activities following spinal surgery [21]: nearly half of them stated distal fusion was a “moderate” to “great deal” factor in allowing sports after scoliosis surgery. In contrast, a more recent survey of high-volume surgeons downplayed.

LIV as a factor for RTS [23]. Considering the type of implants, Lehman et al. [23] in a survey of 23 surgeons using different types of fixation found pedicle screw instrumentation to allow earlier return to noncontact and contact sports when compared to hooks or hybrid constructs [3, 24]. Certainly, stronger 3-column fixation decreases the risk of implant dislodgement and migration [12]. Nevertheless, around 20% of surgeons advise against returning to collision sports, regardless of the instruments used, and the remainder generally suggest a more cautious recovery (at least 12 months) [21, 23].

Many reports agree that it is reasonable to consider the level and extent of spine fusion in the decision to return to full athletic participation, with greater caution being exercised in cases of lower-level fusions [2, 7, 25]. However, according to the analysed studies, > 90% of the patients return to sports within the 1^st^ postoperative year [8, 10–12], regardless of the surgeons’ indications. In particular, Sarwahi et al. [12] report that 33% of the patients returned to their respective sport (contact, non-contact and collision sports) by 12 weeks and 82% by 24 weeks; Tarrant et al. [8] observed that 51.4% of their 70 AIS patients had returned to competitive and contact sports by 24 weeks. Therefore, patients often return to activities much sooner than generally recommended [12]. Even though the vast majority returns to activity, some change sport or decrease intensity [7, 10, 11]: while swimming, horseback riding and athletics were the most popular sports before surgery, gym, cycling and swimming were preferred after surgery [10]; in particular, a decrease has been reported in a number of participants in cheerleading and gymnastics, activities which require a high level of truncal flexibility [7, 10, 11].

There is a paucity of studies that evaluate independent variables that might limit the return to sport after spinal fusion for AIS. Several factors are responsible: decreased flexibility, physical deconditioning, fear of injuries and low back pain have been reported as the most important reasons [7, 10, 20]. Decreased flexibility and functional limitation seem to be strongly related to the distal fusion level: Fabricant et al. [7] and Lonner et al. [9] reported a “stepwise decline” between distal fusion level and rate of return to same or higher level of activities: in particular, the closer the fusion gets to L4, the less likely the patient is to return to sports. On the contrary, some authors [8, 11, 12] didn’t find any factor delaying return to any level of sports: this may be due to the fact that teenagers who are motivated to play sports do not get deterred after surgery and return to playing on their own terms. Infact, some studies [7, 8, 11, 12] reported that in contrast to return to school, many patients return to physical activity much earlier than surgeons recommended. Therefore, it is unknown whether the surgical intervention is actually responsible for some patients changing sports or whether this is due to the natural progression through adolescence, parental influence or some other factors [12].

In conclusion, according to current evidence, patients who received spinal arthrodesis for Adolescent Idiopathic Scoliosis can safely return to any sport, even those that require extreme levels of spinal and pelvic movements such as gymnastics and golf; of course, it is important to make clear that in some cases (especially after long fusions), the loss of mobility could make it difficult for them to play at the same level as preoperatively.

The main limitations of the present review are the paucity of studies that address the topic, the retrospective nature of many of them and the small and non-homogeneous cohorts they sampled. As there is little evidence of the spinal loading that occurs during extreme levels of spinal and pelvic movements, there is lack of scientific evidence-based recommendations or guidelines surgeons and other health care providers can follow. Moreover, the timing of return to sport and other activities can be influenced by a number of factors that none of the studies evaluated, ranging from parental influence to socioeconomic, medical and psychosocial factors. Therefore, further prospective comparative studies, with a longer follow-up period and homogeneous cohorts are needed.
